# Serum lipid profiles and risk of depression: a UK Biobank prospective cohort study

**DOI:** 10.3389/fnut.2026.1839994

**Published:** 2026-05-08

**Authors:** Mengze Du, Chunyu Li

**Affiliations:** 1 Institute of Digestive Disease, The Affiliated Qinqyuan Hospital (Qinqyuan People’s Hospital), Guangzhou Medical University, Qingyuan, Guangdong, China; 2 Laboratory of Neurodegenerative Disorders, Department of Neurology, National Clinical Research Center for Geriatric, West China Hospital, Sichuan University, Chengdu, China

**Keywords:** cohort study, depression, non-linear relationship, serum lipids, UK Biobank

## Abstract

**Background:**

The relationship between serum lipids and depression is poorly characterized, with existing studies yielding inconsistent results. We aim to prospectively investigate the associations between 11 serum lipid traits and risk of incident depression in a large cohort from the UK Biobank.

**Methods:**

The primary exposures were baseline serum concentrations of Apolipoprotein A (ApoA), Apolipoprotein B (ApoB), HDL-C, LDL-C, triglyceride, total cholesterol, total esterified cholesterol, total free cholesterol, total choline, total fatty acids, and Lipoprotein A, while the primary outcome was diagnosis of depression. Cox proportional hazards models were used to calculate hazard ratios (HRs). Non-linearity was assessed using restricted cubic splines.

**Results:**

Over a mean follow-up of 12.3 years, 19,303 participants developed depression among 445,105 participants. In multivariable models, significant inverse associations were observed for ApoB (HR = 0.85, 95% CI = 0.79–0.91), LDL-C (HR = 0.89, 0.86–0.92), and total cholesterol (HR = 0.95, 0.94–0.97), and nominal inverse association for ApoA (HR = 0.91, 0.85–0.98). Quartile analyses showed monotonic decreases in depression risk for each lipid. Restricted cubic spline analyses revealed a U-shaped association for ApoA, indicating elevated depression risk at both extremely low and high concentrations, whereas other lipid traits showed monotonic decreasing relationships. Furthermore, the associations were significantly modified by smoking, with attenuated protective effects among heavy smokers.

**Conclusion:**

These findings suggest maintaining specific lipid levels within a moderate range may be important for mental health, particularly in conjunction with healthy lifestyle factors.

## Introduction

Depression stands as a paramount challenge to global public health, representing one of the leading causes of years lived with disability worldwide (1). Beyond its profound personal suffering, depression exerts a substantial economic burden on healthcare systems and societies at large (2). The pathophysiology of depression is complex, transcending the traditional boundaries of neuroscience to implicate a dynamic interplay between genetic predisposition, psychosocial stressors, and underlying biological vulnerabilities (3). The monoamine hypothesis, which has dominated for decades, is considered insufficient to fully explain the disorder’s heterogeneity, leading to a paradigm shift towards multifactorial models that incorporate neuroinflammation, neuroendocrine dysregulation, and metabolic dysfunction (4). These challenges underscore the critical need for early detection strategies to identify at-risk individuals during preclinical stages, and modifiable risk factors that could inform primary prevention approaches.

A compelling body of evidence has established a robust bidirectional link between depression and cardiometabolic diseases (5, 6). This comorbidity suggests shared biological pathways, such as chronic low-grade inflammation, which is observed in both depressive states and cardiometabolic conditions (7). This intersection has directed scientific inquiry towards biomarkers traditionally associated with cardiovascular health, positing that they may also play an aetiological role in mood disorders. Serum lipids, including lipoproteins, cholesterol, and fatty acids, are fundamental biological molecules with diverse functions that extend from their canonical role in cardiovascular risk to critical processes in the central nervous system (8). Given this biological plausibility, epidemiological studies have investigated the association between serum lipids and depression, but have drawn inconsistent findings. One study among 2,456 middle-aged Finnish men found that low levels of total cholesterol (TC) and low-density lipoprotein cholesterol (LDL-C) are linked to a higher risk of depression (9). Another research has linked elevated triglycerides and low high-density lipoprotein cholesterol (HDL-C) to increased depressive symptoms (10). A 21-year prospective study of 211,200 participants from the Swedish AMORIS cohort reported that high levels of glucose and triglycerides were associated with increased risk of depression, anxiety, and stress-related disorders, while high HDL-C was protective; however, no significant associations were observed for LDL-C or total cholesterol in linear models (11). Conversely, a prospective study of 24,216 postmenopausal women from the Women‘s Health Initiative found that the lowest quintile of LDL-C was associated with a 25% increased risk of developing depressive symptoms (HR = 1.25, 95% CI: 1.05–1.49), with the elevated risk confined to untreated LDL-C levels below 100 mg/dL (12). Yet a cross-sectional study among Indians found null associations between serum lipids and depression (13). These discrepancies may be attributed to several critical factors: (1) inability to establish temporal precedence, leaving the findings vulnerable to reverse causation; (2) inadequate adjustment for a comprehensive set of potential confounders; (3) heterogeneity in outcome definition and ascertainment, (4) population characteristics, and (5) limited sample size in prior studies.

**Figure 1:**
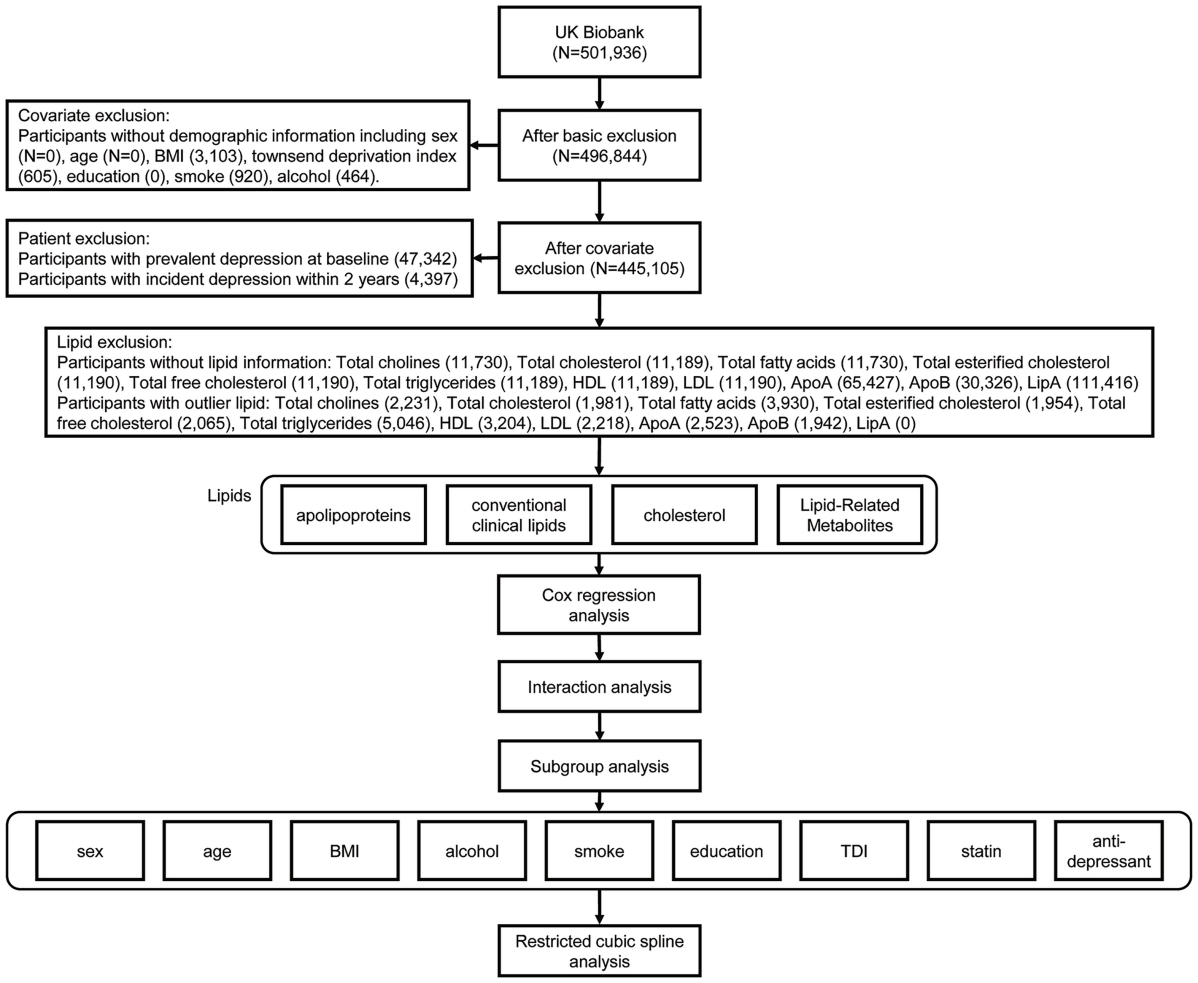
Flowchart of the study.

In this context, this study aims to conduct a thorough prospective investigation of the associations between 11 serum lipid traits and the risk of incident depression based on data from the UK Biobank, a large-scale resource with deep phenotypic and biomarker data (Figure 1). We hypothesize that specific lipid profiles are independently associated with long-term risk of developing depression. The findings will help clarify a contentious area of research and may inform future strategies for the prevention and personalized management of depression.

## Methods

### Participants

This study utilized data from the UK Biobank, a prospective population-based cohort of over half a million adults recruited across 22 UK assessment centers (14). Participants completed comprehensive baseline assessments including questionnaires, physical measurements, blood sampling, genotyping, and electronic health record linkage, with all data collected under NHS ethical approval (16/NW/0274) following written informed consent.

### Exposures

The primary exposures were baseline serum concentrations of Apolipoprotein A (ApoA), Apolipoprotein B (ApoB), high-density lipoprotein cholesterol (HDL-C), low-density lipoprotein cholesterol (LDL-C), triglyceride, total cholesterol, total esterified cholesterol, total free cholesterol, total choline, total fatty acids, and Lipoprotein A. Blood samples were collected from participants at the final station of the baseline visit, and serum was prepared and stored at a temperature of −80 °C until assayed. Following standardized protocols, serum lipid traits were measured by immunoturbidimetric analysis on a Beckman automated haematology analyser (Beckman Coulter AU5800). Lipoprotein A was measured by immunoturbidimetric analysis using the Randox AU5800. Details regarding the assay and quality control procedures could be found in the UK Biobank website.

### Outcomes

The primary outcome we focused on was new diagnosis of depression. Depression cases (including prevalent and incident cases) were identified from multiple sources including participants’ self-reported diagnoses obtained through verbal interviews and electronic health records, which encompassed hospital admissions data, primary care data, and death registers. The coding systems employed were the International Classification of Diseases codes, and the Read coding system codes. The baseline period was defined as the date of recruitment, while the end of the follow-up period was defined as the date of depression incidence, date of death, or end of follow-up, whichever occurred first. The follow-up for depression incidence was conducted until June 15, 2025. To address concerns regarding reverse causality, we excluded participants with prevalent depression at the time of study enrollment, and those with a short latency period of 2 years or less between the initial sampling and depression diagnosis.

### Statistical analysis

We analyzed the associations between baseline levels of each lipid trait and depression risk using Cox proportional hazards models. Our analyses were conducted initially for the entire study population, and then stratified by different groups based on key covariate. Interaction analyses were performed by incorporating interaction terms into the Cox models. We utilized two models for our analyses. In Model 1, the minimally adjusted model, we adjusted for the fundamental demographic variables of sex and age. In Model 2, the fully adjusted model, we included additional covariates to account for socioeconomic status (Townsend deprivation index and education) lifestyle factors (body mass index, smoking status, alcohol consumption) (Supplementary methods), and medication use (statin and antidepressants) (Supplementary Tables 1, 2). To ensure the robustness and accuracy of our results, we excluded participants with missing values for any of the variables included in the models, as well as samples with lipid values exceeding 3 standard deviations away from the mean. We further conducted a sensitivity analysis excluding the cases that were identified only by self-report without corresponding hospital or primary care records, to exclude potential self-report bias. A *p* value below 0.005 (0.05/11) was considered statistically significant after the Bonferroni correction. Statistical analyses were performed in R v3.5.3.

## Results

### Population characteristics

A total of 445,105 individuals remained for regression analysis after applying the exclusion criteria, including 208,436 males (46.8%) (Figure 1). During a mean follow-up of 12.3 years, 19,303 participants developed incident depression. Using a hierarchical classification, these cases were distributed as follows: 15,461 from hospital admissions; 2,686 from primary care without a hospital record; 1,135 from self-report only; and 21 from death registries. Baseline characteristics of participants who developed incident depression versus those who did not are shown in Table 1. Absolute differences between the two groups were small, with standardized mean differences <0.1 for most continuous variables.

**Table 1:** Baseline characteristics of the UK Biobank analytical cohort by disease status.

Baseline characteristic	Cases	Non-cases	Standardized mean differences
Sociodemographic			
Age (years)	56.24 (8.40)	56.68 (8.09)	−0.054
Sex (male)	0.39 (7487)	0.47 (200949)	0.034
BMI (kg/m^2^)	28.31 (5.43)	27.29 (4.66)	0.217
Townsend index	−0.72 (3.29)	−1.40 (3.04)	0.224
Education			
High	0.84 (16173)	0.90 (384891)	0.045
Medium	0.03 (635)	0.03 (11251)	0.008
Low	0.13 (2495)	0.07 (29660)	0.047
Smoke			
No	0.26 (5072)	0.34 (146562)	0.035
Occasionally	0.33 (6323)	0.32 (137740)	0.002
Mostly	0.41 (7908)	0.33 (141500)	0.033
Alcohol			
Never	0.23 (4530)	0.20 (85350)	0.017
Special occasions	0.77 (14773)	0.80 (340452)	0.017
1–3 times a month	0.13 (2511)	0.03 (11559)	0.120
1–2 times a week	0.87 (16792)	0.97 (414243)	0.120
3–4 times a week	0.11 (2113)	0.07 (31666)	0.027
Daily	0.15 (2869)	0.11 (46732)	0.025
Statin use			
Yes	0.12 (2392)	0.11 (46622)	0.009
No	0.25 (4802)	0.26 (111383)	0.006
Antidepressant use			
Yes	0.19 (3637)	0.24 (101318)	0.024
No	0.18 (3490)	0.21 (88081)	0.013
Lipids			
Total choline	2.60 (0.42)	2.60 (0.40)	−0.008
Total cholesterol	4.63 (0.94)	4.69 (0.93)	−0.061
Total fatty acids	12.24 (2.40)	12.14 (2.33)	0.044
Total esterified cholesterol	3.36 (0.68)	3.41 (0.67)	−0.064
Total free cholesterol	1.27 (0.27)	1.28 (0.26)	−0.053
Triglyceride	1.35 (0.57)	1.31 (0.56)	0.067
HDL-C	1.32 (0.33)	1.33 (0.32)	−0.048
LDL-C	1.75 (0.44)	1.77 (0.43)	−0.062
Apolipoprotein A	1.52 (0.27)	1.53 (0.26)	−0.053
Apolipoprotein B	1.02 (0.24)	1.03 (0.23)	−0.034
Lipoprotein A	44.13 (49.09)	44.63 (49.17)	−0.010

Baseline characteristics were presented as numbers (percentage) for categorical variables and as mean (standard deviation) for continuous variables. Standardized mean differences (Cohen’s *d*) for continuous variables and Cramér’s *V* for categorical variables are reported.

### Association of serum lipid traits with depression

**Figure 2:**
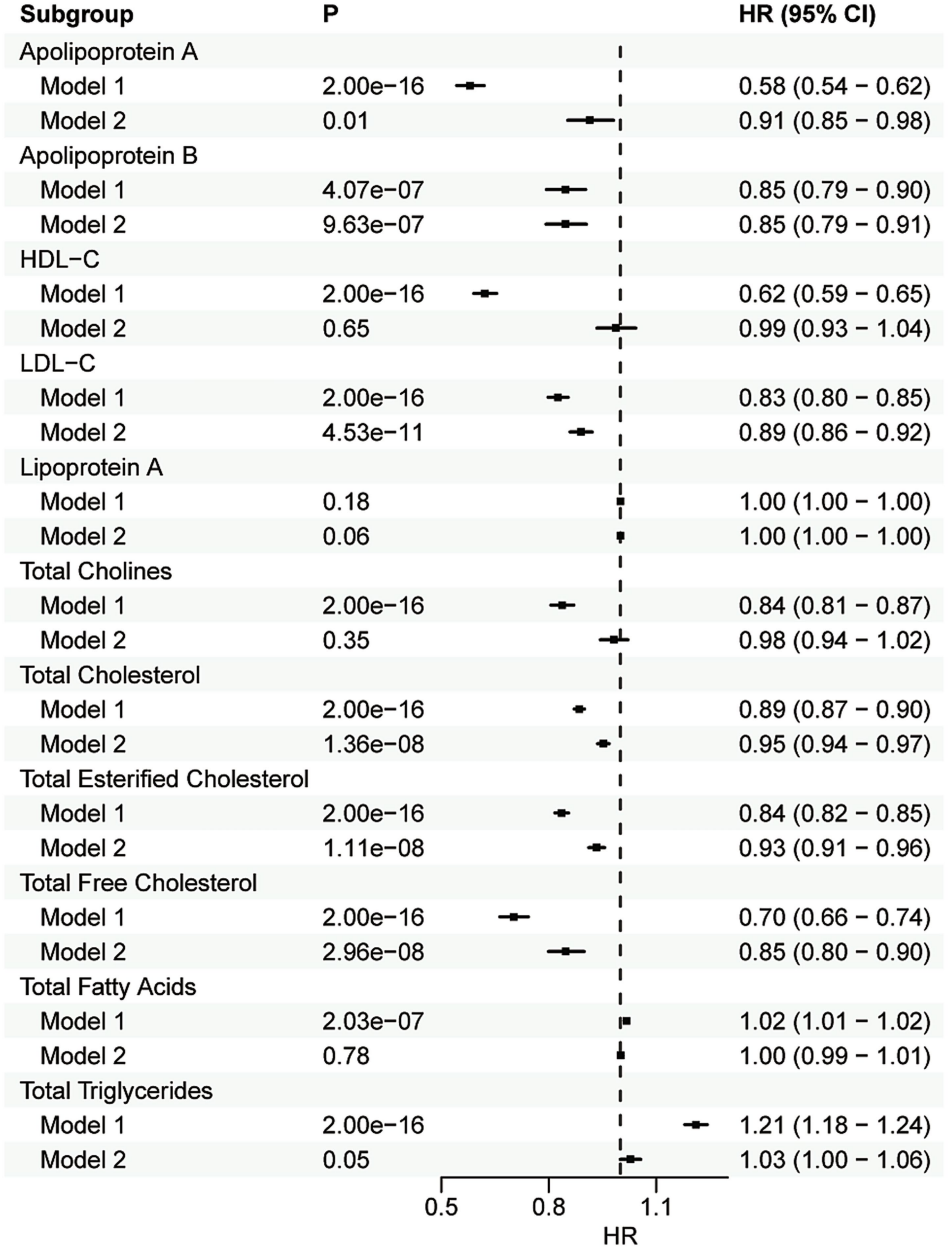
Forest plot showing the results from the Cox regression analysis. Results from Cox proportional hazards regression analysis of the association between each lipid and depression. Error bars indicate 95% confidence intervals.

In the initial model (Model 1), depression was significantly associated with all the lipid traits except Lipoprotein A (Figure 2). After further adjustment (Model 2), inverse associations with incident depression remained for ApoB (HR = 0.85, *p* = 9.63E−07, 95% CI = 0.79–0.91), LDL-C (HR = 0.89, *p* = 4.53E−11, 95% CI = 0.86–0.92), total cholesterol (HR = 0.95, *p* = 1.36E-08, 95% CI = 0.94–0.97), total esterified cholesterol (HR = 0.93, *p* = 1.11E−08, 95% CI = 0.91–0.96), and total free cholesterol (HR = 0.85, *p* = 2.96E−08, 95% CI = 0.80–0.90). In addition, nominal association was identified for ApoA (HR = 0.91, *p* = 0.01, 95% CI = 0.85–0.98) (Figure 2). In the sensitivity analysis, the identified associations remained for ApoB (HR = 0.82, *p* = 3.47E−08, 95% CI = 0.77–0.88), LDL-C (HR = 0.87, *p* = 2.32E−13, 95% CI = 0.84–0.91), total cholesterol (HR = 0.94, *p* = 6.73E−11, 95% CI = 0.93–0.96), total esterified cholesterol (HR = 0.92, *p* = 4.55E−11, 95% CI = 0.90–0.94), total free cholesterol (HR = 0.82, *p* = 1.47E−10, 95% CI = 0.77–0.87), and ApoA (HR = 0.90, *p* = 5.10E−03, 95% CI = 0.84–0.97).

**Figure 3:**
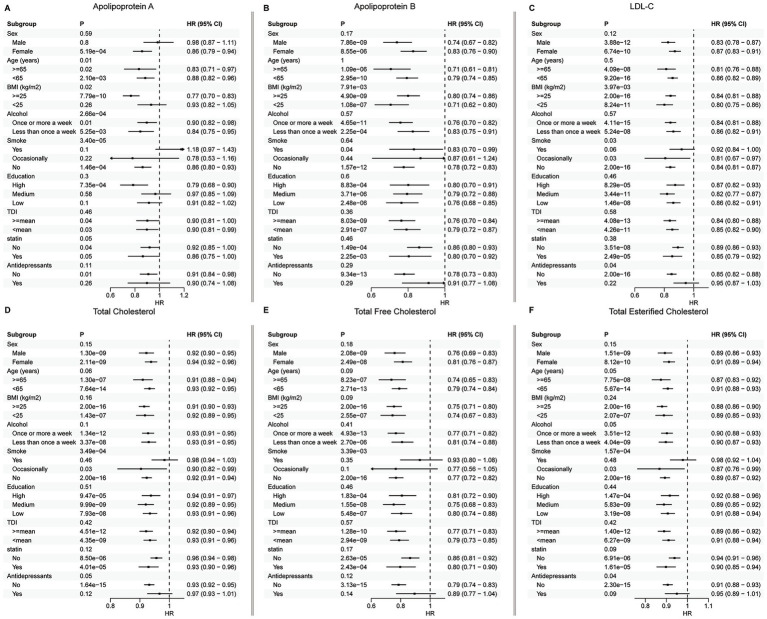
Subgroup analyses of the association between lipid traits and depression. Forest plots showing results from Cox proportional hazards regression analyses of the association between lipid traits and depression across subgroups: **(A)** apolipoprotein A, **(B)** apolipoprotein B, **(C)** LDL cholesterol, **(D)** total cholesterol, **(E)** total free cholesterol, and **(F)** total esterified cholesterol. Error bars indicate 95% confidence intervals. The HR axis is truncated, with arrows indicating values beyond the displayed range.

We identified significant interaction between smoking and several lipid traits, including ApoA, LDL-C, total cholesterol, total free cholesterol, and total esterified cholesterol (Figure 3). Subgroup analyses demonstrated that the effect of these lipids was more pronounced among lighter smokers. Significant interaction was also observed between ApoA and alcohol consumption (*p* = 1.07E−06), with a stronger protective effect of ApoA among lighter drinkers, although both groups showed statistically significant associations (Figure 3). Additionally, significant interaction was identified between BMI and several lipid traits. Specifically, the protective effects of ApoA was more evident in individuals with higher BMI, while the protective effects of ApoB and LDL-C were more pronounced in those with lower BMI. Subgroup analyses otherwise demonstrated largely consistent trends across most demographic strata (Figure 3).

**Figure 4:**
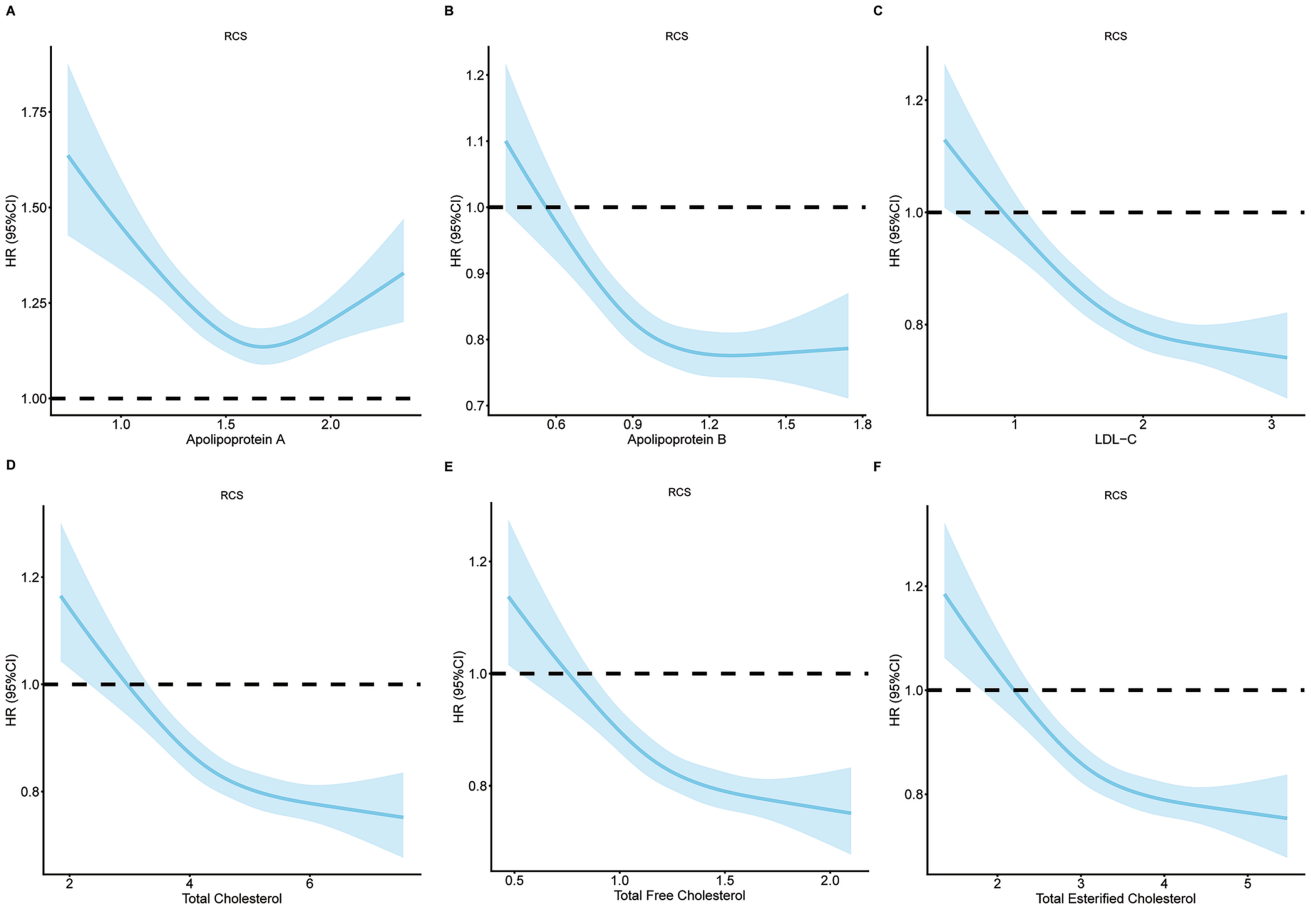
Non-linear association between lipid traits and depression.Multivariable restricted cubic spline analyses showing hazard ratios for depression associated with **(A)** Apolipoprotein A, **(B)** Apolipoprotein B, **(C)** LDL cholesterol, **(D)** total cholesterol, **(E)** total free cholesterol, and **(F)** total esterified cholesterol. The solid line represents the hazard ratio, and the shaded area is the 95% confidence interval.

To further characterize the dose–response relationship, we employed restricted cubic splines. These analyses revealed that the association between lipid traits and depression risk varied by lipid type. Specifically, a U-shaped pattern was observed for ApoA, indicating elevated risk at both low and high extremes. For the remaining lipids (including ApoB, LDL-C, and total cholesterol), the relationships were monotonic decreasing, with higher lipid levels associated with lower depression risk across the observed range (Figure 4).

**Figure 5:**
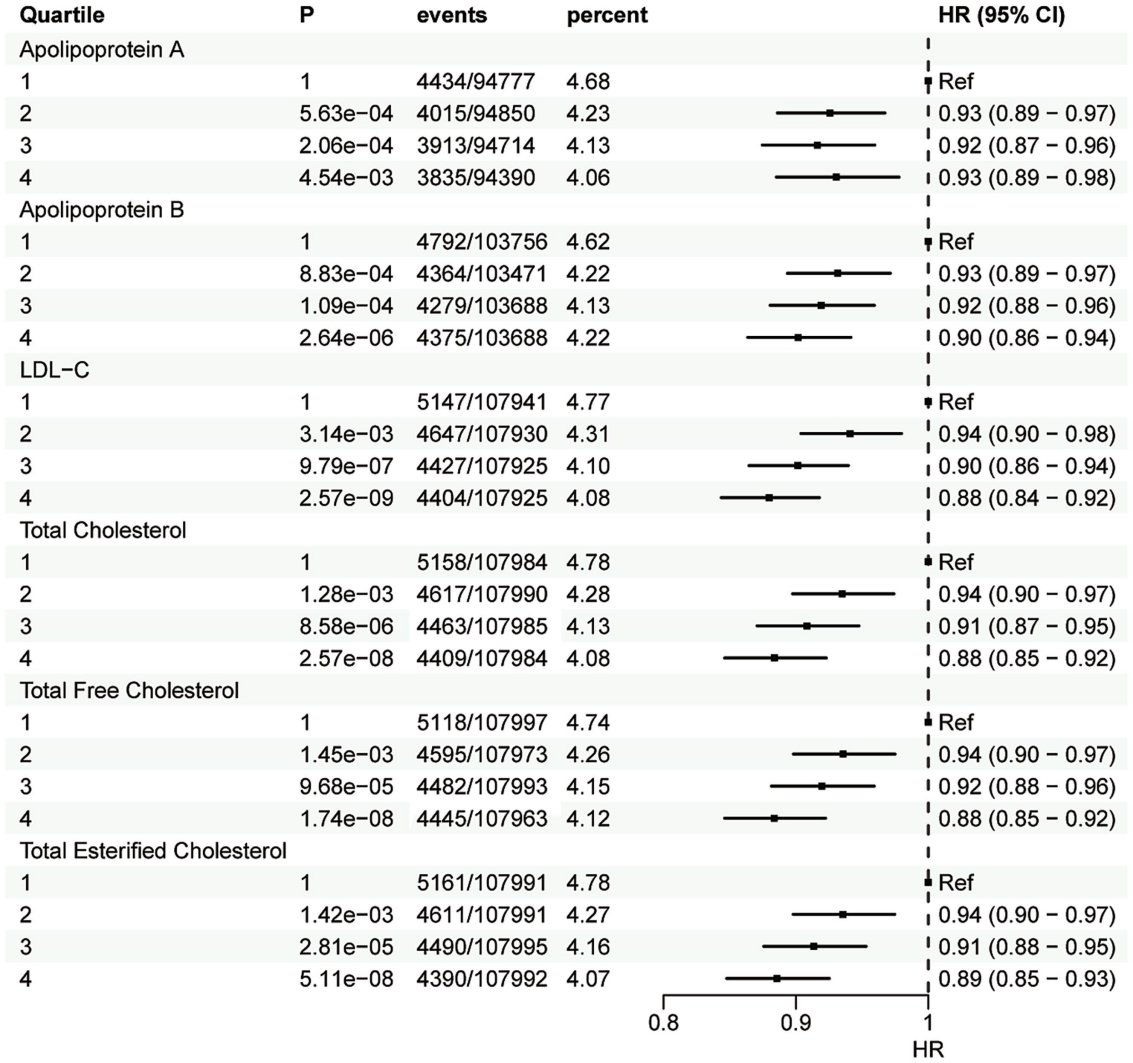
Hazard ratios of the association between quartiles of lipids and depression. HR, hazard ratio; No., number. Hazard ratios are relative to the lowest quartile (Q1).

Quartile analysis revealed a strong monotonic decrease in depression incidence with ascending quartiles of total cholesterol (Q1: 4.78%, Q4: 4.08%), total esterified cholesterol (Q1: 4.78%, Q4: 4.07%), total free cholesterol (Q1: 4.74%, Q4: 4.12%), LDL-C (Q1: 4.77%, Q4: 4.08%), ApoA (Q1: 4.68%, Q4: 4.06%), ApoB (Q1: 4.62%, Q4: 4.22%) (Figure 5).

## Discussion

In this large-scale prospective cohort study, we delineate a complex relationship between serum lipid levels and depression risk. While initial analyses suggested monotonic inverse associations for a broad spectrum of lipids, including ApoB, LDL-C, total cholesterol, and ApoA, more sophisticated modelling revealed that this relationship is not uniform across lipid traits. Specifically, a U-shaped association was observed for ApoA, whereas the other lipids exhibited monotonic decreasing relationships. Furthermore, we demonstrate that this relationship is not static but dynamically modified by lifestyle, with smoking significantly attenuating the protective association of these lipids. These findings collectively argue for a paradigm shift in how we conceptualize the role of lipids in brain health, moving beyond a cardiometabolic lens to appreciate their distinct neurobiological functions.

The central nervous system is the most cholesterol-rich organ in the body, and this cholesterol is indispensable for its structural and functional integrity (15). It is not merely a passive structural component but a dynamic regulator of synaptic function. Cholesterol within the lipid rafts of neuronal membranes is crucial for the assembly and function of neurotransmitter receptors, including those for serotonin, thereby directly modulating the synaptic signaling implicated in depression pathophysiology (16). Apolipoproteins like ApoA and ApoB are crucial for cholesterol transport and redistribution in the brain, which in turn influences synaptic plasticity and repair. They form lipoproteins that deliver essential lipids to neurons and support their function (17). Therefore, sufficient systemic availability of these lipid precursors may be crucial for maintaining optimal brain structure and function, thereby conferring resilience against the development of depression.

The robust protective associations observed for this specific lipid panel illuminate a complex network of neurobiological pathways, moving beyond a singular mechanism to a multi-system view of lipid-mediated resilience. The strong inverse relationship for ApoB and its correlated trait, LDL-C, underscores the critical importance of exogenous cholesterol delivery for neuronal structure and function. The brain, while capable of *de novo* cholesterol synthesis in astrocytes, relies on the efficient trafficking of cholesterol to distant neurons via ApoB-containing lipoproteins for synaptogenesis, axonal growth, and the maintenance of intricate synaptic architecture, particularly in highly plastic regions like the hippocampus and prefrontal cortex that are vulnerable to stress and depression (8, 18). Our findings suggest that insufficient ApoB may limit this vital delivery system, impairing the brain’s capacity for adaptive plasticity and repair. This is complemented by the associations observed for total cholesterol and its fractions. The pronounced effect of total free cholesterol highlights its role not merely as a passive structural lipid but as a dynamic modulator of membrane microdomains. By determining membrane fluidity, free cholesterol regulates the function of integral proteins critical to neurotransmission, thereby directly influencing the neurochemical environment implicated in depression (19, 20). In contrast, the more modest protective association for ApoA likely operates through a distinct, primarily homeostatic and protective pathway. ApoA, the principal component of HDL, is a key mediator of reverse cholesterol transport and possesses potent anti-inflammatory, antioxidant, and vasoprotective properties (21). In the cerebral context, ApoA has been shown to suppress endothelial activation, inhibit the adhesion of pro-inflammatory monocytes to the cerebrovasculature, and help preserve the integrity of the blood–brain barrier (22, 23). This is critical, as a leaky blood–brain barrier allows the influx of peripheral cytokines and immune cells, driving the neuroinflammation consistently observed in major depressive disorder (24). Furthermore, HDL and ApoA have been demonstrated to directly mitigate the neurotoxic effects of *β*-amyloid and dampen microglial activation *in vitro*, suggesting a direct neuroprotective role that extends beyond vascular health (22). Collectively, these associations paint a picture where ApoB/LDL-C supports active neural building and remodeling, free cholesterol ensures optimal synaptic communication, and ApoA maintains a healthy cerebral environment by quelling inflammation and protecting vascular integrity. A deficiency in any of these interlinked systems may compromise the brain’s structural and functional resilience, thereby increasing vulnerability to depression.

The robust inverse associations observed for total cholesterol, and more distinctly for its esterified and free fractions, underscore the fundamental importance of systemic cholesterol homeostasis in neurobiological resilience. Total cholesterol serves as a summary measure of the body’s cholesterol burden, but the divergent effect sizes for its components reveal a more nuanced story. The particularly strong protective association with total free cholesterol likely reflects its direct and indispensable role in the central nervous system, where it is incorporated into neuronal membranes and myelin sheaths to maintain structural integrity, regulate membrane fluidity, and facilitate efficient synaptic transmission by influencing the function of neurotransmitter receptors (17). In contrast, total esterified cholesterol, which represents the storage and transport form of cholesterol, may serve as a readily available pool to support these dynamic processes, including synaptogenesis and repair in response to stress (8). The finding that free cholesterol exhibited the most potent association suggests that the brain’s vulnerability to depression may be particularly sensitive to the availability of this biologically active, unesterified form, which is immediately utilizable for critical neuronal functions over the more inert, esterified reservoir. This delineation implies that the protective effect is not merely a passive consequence of overall cholesterol levels but is actively mediated by the specific metabolic partitioning of cholesterol into functional pools that directly support neuronal health and plasticity (19).

Non-linear associations between lipid-related biomarkers and depression have been reported in previous studies. For example, a U-shaped association was identified between LDL-C and severe depression among men in the US National Health and Nutrition Examination Survey (NHANES) (25), and between total cholesterol and longitudinal increase in somatic complaints among US adults (26). More recently, nonlinear threshold associations have been reported for the non-HDL-C/HDL-C ratio (NHHR) with late-life depression (27) and for the Lipid Accumulation Product (LAP) with depression prevalence among middle-aged and elderly men (28). These studies suggest that non-linear relationships between lipid markers and depression are not unprecedented. However, no previous studies have reported non-linear association between ApoA and depression. In our study, a U-shaped association was observed between ApoA and depression, indicating that both deficient and excessive levels are detrimental to mental health. Low ApoA levels may impair neuronal maintenance and synaptic signalling, as ApoA-I possesses potent anti-inflammatory, antioxidant and immunomodulatory properties (29). Reduced ApoA-I has been repeatedly reported in major depressive disorder and is associated with compromised reverse cholesterol transport and heightened systemic inflammation (29, 30). Although high ApoA levels are generally considered protective, emerging evidence suggests that in the context of chronic inflammation, HDL and ApoA1 may become dysfunctional, losing their anti-inflammatory properties and potentially even acquiring pro-inflammatory effects (29). This could explain why extremely high ApoA levels might paradoxically increase depression risk. For other lipid traits (ApoB, LDL-C, and total cholesterol), the relationships were monotonic decreasing, with no evidence of elevated risk at higher levels. The discrepancy between the monotonic quartile trends and the U-shaped spline curves arises because the ascending limb of the U-shape is confined to the extreme upper tail. The highest quartile averages these extreme values with moderate-high levels that remain protective, thus masking the elevated risk at the very top. This underscores the importance of non-linear modelling to detect risk at the tails of the distribution.

The relationship between lipid levels and depression risk is context-dependent, as evidenced by its modification by smoking status. This indicates that the neuroprotective potential of lipids can be compromised by exposure to potent pro-inflammatory environmental stressors. The attenuated protective effect of lipids in heavy smokers could be attributed to smoking’s profound pro-inflammatory and oxidative stress effects, which might overwhelm any beneficial neurobiological influence of lipids (31). Alternatively, it may also directly modify lipid particles, making them less functional or more pro-inflammatory, thereby decoupling serum lipid levels from their beneficial neurotrophic effects (32). This underscores that the psychiatric implication of a biomarker cannot be viewed in isolation but is contingent upon an individual’s broader lifestyle and metabolic context.

Ultimately, our findings introduce a novel conceptual model that synthesizes the historically discordant literature on lipids and depression. The inconsistencies among prior studies, which reported negative, null, or positive associations (10, 33, 34), could not be viewed merely as contradictory. Instead, they appear as predictable fragments of a more complex, non-linear reality. While discrepancies could be partly attributed to variations in study design, sample characteristics, and confounder adjustment (35), the discovery of a consistent U-shaped relationship offers a unifying pathophysiological explanation. This model elucidates how a linear analytical approach applied to a fundamentally non-linear association would inevitably yield a net null result when opposing risks at the extremes cancel each other out (36), and would produce seemingly protective or detrimental findings depending on the lipid distribution within a specific cohort. Consequently, our results transcend these apparent contradictions by demonstrating that the association is not fixed but is dynamically shaped by an individual’s position on the non-linear risk continuum and their exposure to effect-modifying factors. This reframing not only reconciles prior evidence but also establishes a new foundation for future research, shifting the question from whether lipids are associated with depression to under what specific physiological and contextual conditions this association manifests.

The major strengths of this study include its prospective design, the large sample size, the long-term follow-up, the measurement of a wide array of lipid traits, and the rigorous adjustment for a comprehensive set of potential confounders. The use of multiple analytical approaches allowed us to characterize the associations with greater depth and precision. However, several limitations must be acknowledged. First, despite extensive adjustment for demographic, lifestyle, and medication use, residual confounding cannot be entirely ruled out due to the lack of detailed information on key variables, such as medication dosage, duration, adherence, or other unmeasured or imprecisely measured confounders. Second, the diagnosis of depression was based on health records and self-report, which may miss milder cases not seeking clinical care, potentially leading to outcome misclassification. Third, lipid levels were measured only at baseline, and changes over time were not captured. Fourth, the UK Biobank participants are not fully representative of the general population, which may limit the generalizability of our findings.

## Conclusion

This study demonstrates that the association between serum lipids and depression differs across lipid traits. Higher levels of ApoB, LDL-C, and total cholesterol were consistently associated with lower depression risk, supporting a protective role. In contrast, ApoA showed a U-shaped relationship, with elevated risk at both low and high concentrations, indicating that maintaining ApoA within a moderate range may be optimal for mental health. These findings challenge the assumption of uniform linear effects and highlight the need for lipid-specific risk assessment. Future research should replicate these observations, investigate underlying mechanisms, and explore the potential of lipid profiling in depression prevention.

## Data Availability

The original contributions presented in the study are included in the article/Supplementary material, further inquiries can be directed to the corresponding author.
