# Supplementary material for: Serum lipid profiles and risk of depression: a UK Biobank prospective cohort study
Source: Front Nutr. 2026 May 8;13:1839994. doi: 10.3389/fnut.2026.1839994 (PMC13193863; doi:10.3389/fnut.2026.1839994)
Supplement: Supplementary file 1 [file Data_Sheet_1.docx]

**Supplementary Table 1. Statins contained within the UK Biobank self-reported medication data and their associated medication codes.**

| **Drug** | **Mapped UK Biobank Codes** |
| --- | --- |
| simvastatin | 1140861958, 1140910652, 1140881748, 1141200040, 1141188146, 1140910654 |
| pravastatin | 1140888648, 1140910632, 1140861970 |
| fluvastatin | 1140888594, 1140864592 |
| atorvastatin | 1141146234, 1141146138 |
| rosuvastatin | 1141192410, 1141187780, 1141192414 |
| velastatin | 1140910654 |
| eptastatin | 1140910632 |

Each medication code, from Data-coding 4, represents an individual drug that was mapped to the generic name for the statin.

**Supplementary Table 2. Antidepressants contained within the UK Biobank medication data and their associated medication codes.**

| **Classes** | **Drug** | **Mapped UK Biobank Codes** |
| --- | --- | --- |
| SSRIs | citalopram, dapoxetine, escitalopram, fluoxetine, fluvoxamine, paroxetine, sertraline | 1140921600, 1141180212, 1140879540, 1140879544, 1140867888 |
| SNRIs | duloxetine, venlafaxine | 1141200564 |
| SARIs | nefazodone, trazodone | 1140917460 |
| TCAs | amitriptyline, amoxapine, clomipramine, dosulepin, doxepin, imipramine, lofepramine, nortriptyline, protriptyline, trimipramine | 1140879616, 1140867774, 1140879620, 1140909806, 1140867640, 1140879630, 1140867726, 1140867818, 1140879632 |
| TeCAs | maprotiline, mianserin, mirtazapine | 1140879552, 1140879556 |
| Others | agomelatine, amitriptyline_perphenazine, bupropion, flupentixol, isocarboxazid, moclobemide, nortriptyline_fluphenazine, phenelzine, reboxetine, tranylcypromine, tranylcypromine_trifluoperazine, viloxazine, vortioxetine | 1141176854, 1140909800, 1140867856, 1140867920, 1140867850, 1141151978, 1140867914, 1140879688 |

Each medication code, from Data-coding 4, represents an individual drug that was mapped to the generic name for the antidepressants. Notably, some listed drugs do not have corresponding UK Biobank medication codes and were therefore not captured in the dataset.
