# Supplementary material for: Serum lipid profiles and risk of depression: a UK Biobank prospective cohort study
Source: Front Nutr. 2026 May 8;13:1839994. doi: 10.3389/fnut.2026.1839994 (PMC13193863; doi:10.3389/fnut.2026.1839994)
Supplement: Supplementary file 2 [file Data_Sheet_2.DOCX]

Supplementary Methods

**Details on sociodemographic and lifestyle measures**

Age (data field: 21022): Age at recruitment is a derived variable based on date of birth and date of attending an initial Assessment Center. It refers to the age of the participant on the day they attended an initial Assessment Center, truncated to whole year.

Sex (data field: 31): Sex of participant is acquired from central registry at recruitment.

BMI (data field: 21001): BMI value is constructed from height and weight measured during the initial Assessment Center visit. Value is not present if either of these readings were omitted.

Townsend deprivation index (data field: 22189): Neighborhood-level socioeconomic status was measured using the Townsend index of material deprivation. Townsend deprivation index calculated immediately prior to participant joining UK Biobank. Based on the preceding national census output areas. Each participant is assigned a score corresponding to the output area in which their postcode is located.

Education level (data field: 6138): Education level were transformed into the three categories: “High”: “College or University degree”; “Medium”: “A levels/AS levels or equivalent”, “NVQ or HND or HNC or equivalent”, “Other professional qualifications eg: nursing, teaching”; Low: “O levels/GCSEs or equivalent”, “CSEs or equivalent”, “None of the above”.

Current tobacco smoking (data field: 1239): Participants were asked about their current tobacco smoking status. Possible answers were reordered to 0 (No), 1 (Only occasionally), and 2 (Yes, on most or all days). Current tobacco smoking status was treated as a categorical variable. Responses of “prefer not to answer” were excluded.

Alcohol frequency (data field: 1558): Individuals were asked by the touchscreen questionnaire: “About how often do you drink alcohol?” and could respond with, “Never”, “Special occasions only”, “One to three times a month”, “Once or twice a week”, “Three to four times a week” or “Daily or almost daily”. If participants felt that this value varied, they were instructed to give an average over the last year. Responses of “prefer not to answer” were excluded.
